# Modulation of IL-1β and TGF-β1 Gene Expression in Stress-Induced Depression Rat Supplemented with Malaysian Acacia Honey

**DOI:** 10.3390/foods14223895

**Published:** 2025-11-14

**Authors:** Anis Syamimi Mohamed, Hussin Muhammad, Nik Aina Syazana Nik Zainuddin, Nur Liana Md Nasir, Mohd Rahimi Ashraf Abd Rahman, Lau Mei Siu, Abdah Md Akim, Kalavathy Ramasamy, Mizaton Hazizul Hasan, Zolkapli Eshak

**Affiliations:** 1 National Poison Centre, Main Campus, Universiti Sains Malaysia, Penang 11800, Pulau Pinang, Malaysia; asyamimi@usm.my; 2 Herbal Medicine Research Centre, Institute for Medical Research, Setia Alam 40170, Selangor, Malaysia; hussin.m@moh.gov.my (H.M.); nikaina@moh.gov.my (N.A.S.N.Z.); nurliana.md@moh.gov.my (N.L.M.N.); rahimi.ashraf@moh.gov.my (M.R.A.A.R.); laums@moh.gov.my (L.M.S.); 3 Department of Biomedical Sciences, Faculty of Medicine and Health Sciences, Universiti Putra Malaysia, Serdang 43400, Selangor, Malaysia; abdah@upm.edu.my; 4 Department of Pharmacology and Life Sciences, Puncak Alam Campus, Faculty of Pharmacy, Universiti Teknologi MARA (UiTM), Bandar Puncak Alam, Shah Alam 42300, Selangor, Malaysia; kalav922@uitm.edu.my (K.R.); mizaton_hazizul@uitm.edu.my (M.H.H.)

**Keywords:** Acacia honey, chronic unpredictable mild stress, hyperglycemia, oxidative stress, inflammation

## Abstract

Chronic stress is a key risk factor for depression and metabolic dysfunction, widely mediated through oxidative stress and inflammatory pathways. Natural products such as honey are increasingly investigated for their potential to attenuate stress-induced pathophysiological changes. This study evaluated the protective effects of Malaysian Acacia honey (AH) on glucose regulation, oxidative damage, histopathological alterations, and pro-inflammatory cytokine expression in stress-induced rats. Male Sprague–Dawley rats (n = 42) were subjected to chronic unpredictable mild stress and supplemented with AH, amitriptyline (AMT), or their combination for 28 days. Blood glucose levels, erythrocyte hemolysis, histological changes in liver and kidney, and expression of IL-1β and TGF-β1 in ileum, caecum, and hypothalamus were assessed. Data were reported as mean and standard error of mean (SEM) after three or more independent experiments had been conducted. The data were analyzed using a paired-*t*-test or a one-way or two-way analysis of variance (ANOVA) and considered significant if *p* < 0.05. Stress markedly elevated glucose levels (7.97 ± 0.20 mmol/L), increased hemolysis (14.30% ± 2.96), and induced hepatic (cytoplasmic vacuolation, 1.40 ± 0.25; cell lining absent, 1.20 ± 0.37) and renal lesions (dilated intertubular capillaries, 1.40 ± 0.51; inflammation, 2.20 ± 0.20), accompanied by upregulation of *IL-1β* (1.27-fold ± 0.20) and *TGF-β1* (1.00-fold ± 0.08). Supplementation with AH significantly reduced hyperglycemia, inhibited hemolysis, ameliorated tissue damage, and downregulated pro-inflammatory cytokines. Combination therapy with AH and AMT produced the most significant improvements near to normal level, suggesting synergistic benefits. These findings highlight the therapeutic potential of AH as a natural adjunct in managing stress-related metabolic and inflammatory disturbances.

## 1. Introduction

Chronic stress is a major contributor to the onset and progression of depression and its associated comorbidities, including metabolic disorders, cardiovascular disease, and neurodegeneration. Stress exerts systemic effects primarily through activation of the hypothalamic–pituitary–adrenal (HPA) axis, resulting in hypercortisolemia, impaired glucose homeostasis, and immune dysregulation [1]. Long-term stress has been shown to increase oxidative stress, promote inflammatory responses, and trigger histopathological changes in vital organs such as the liver, kidney, and brain, thereby exacerbating disease risk [2,3].

Oxidative stress plays a central role in the pathophysiology of stress-induced disorders. Overproduction of reactive oxygen species (ROS) and impaired antioxidant defenses lead to lipid peroxidation, protein oxidation, and DNA damage [4]. Similarly, chronic stress promotes inflammation, often characterized by upregulation of pro-inflammatory cytokines such as interleukin-1 beta (IL-1β) and transforming growth factor beta 1 (TGF-β1) [5]. These cytokines have been implicated in both peripheral organ dysfunction and central nervous system alterations, contributing to behavioral and physiological manifestations of stress-related depression [6,7].

Current pharmacological treatments for depression, such as tricyclic antidepressants including amitriptyline (AMT), primarily target monoaminergic pathways [8]. While effective for mood regulation, these drugs often fail to fully address stress-induced oxidative and inflammatory processes and are associated with side effects. This gap highlights the need for adjunctive strategies that target broader pathophysiological mechanisms.

**Figure 1:**
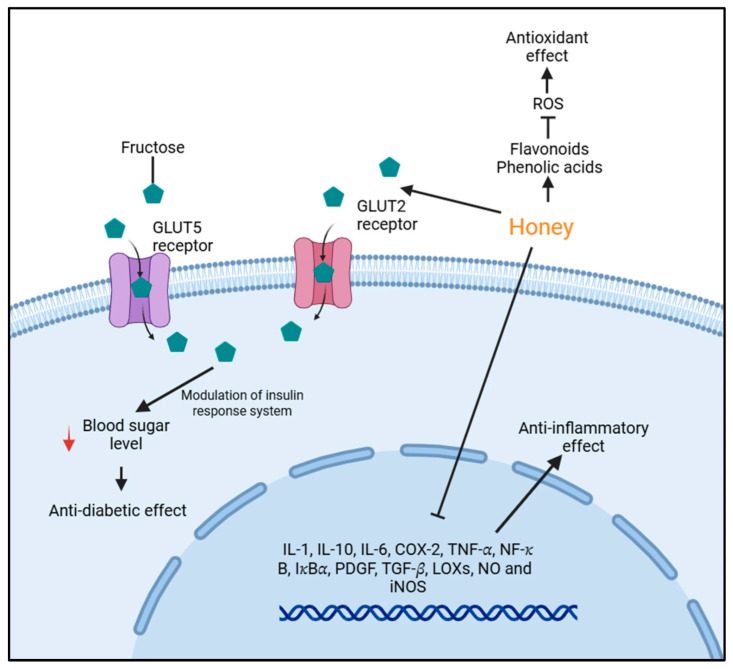
Summary of mechanisms of honey in modulating glucose metabolism and inflammation as reported by Ahmed et al. [20]. Honey contains bioactive compounds such as flavonoids and phenolic acids that exert antioxidant effects by reducing reactive oxygen species (ROS). These compounds also contribute to anti-inflammatory activity by downregulating pro-inflammatory mediators (IL-1, IL-10, IL-6, COX-2, TNF-α, NF-κB, IκBα, PDGF, TGF-β, LOXs, NO, and iNOS). Fructose in honey is transported into cells via GLUT5 and GLUT2 receptors, influencing insulin response and lowering blood glucose levels (red arrow), which may lead to anti-diabetic effects.

Honey, a natural substance produced by bees, is rich in phenolic compounds, flavonoids, and sugars (fructose, glucose, sucrose, maltose and others) that have strong antioxidants and anti-inflammatory properties (Figure 1). Several studies have reported the beneficial effects of honey in mitigating oxidative stress, improving glycemic control, and modulating immune responses [9,10,11]. In animal models, honey supplementation has been shown to lower fasting blood glucose, enhance antioxidant enzyme activities, and protect against oxidative tissue injury [10,12]. Clinical studies further suggest that honey improves antioxidant biomarkers and reduces fasting blood sugar in humans [13].

Malaysian Acacia honey (AH) is a unifloral honey predominantly derived from the nectar of *Acacia mangium* or *Robinia pseudoacacia* trees, known for its distinctive chemical composition as well as high antioxidant capacity due to its flavonoid and phenolic content [14]. A physicochemical analysis revealed that AH contains approximately 39.9% fructose, 30.7% glucose, 4.7% sucrose, and 3.1% maltose, resulting in a fructose-to-glucose ratio of 1.3, which contributes to its relatively low glycemic index and slower crystallization rate [15]. Fructose intake from honey has been reported to attenuate hyperglycemia in various experimental and clinical models, including rodents, healthy individuals, and patients with diabetes [16]. This effect is primarily attributed to delayed gastric emptying, which reduces gastric motility and slows the rate of intestinal glucose absorption, thereby improving postprandial glycemic response [16].

Several studies have investigated the effects of AH intake on blood glucose regulation. For example, Sukri et al. [17] demonstrated that consumption of AH before and during exercise did not significantly alter plasma glucose or insulin levels compared to a commercial sports drink, but it did enhance antioxidant status. Similarly, Ahmad et al. [18] reported that a sodium-enriched AH drink consumed during rehydration after exercise maintained stable blood glucose levels and improved plasma volume recovery. Previous research demonstrated that AH supplementation improves oxidative balance, reduces lipid peroxidation, and enhances immune function [14,19]. However, its effects on stress-induced metabolic, inflammatory, and histopathological changes remain underexplored.

The chronic unpredictable mild stress (CUMS) model employed in this study is a widely accepted paradigm for inducing depression-like behaviors in rodents, including anhedonia, behavioral despair, and neuroinflammatory changes, thereby closely mimicking the core symptoms and pathophysiology of human major depressive disorder (MDD) [21]. The model demonstrates strong face, construct, and predictive validity, making it a valuable tool for investigating the biological mechanisms underlying stress-induced depression and for evaluating potential therapeutic interventions [22]. Sprague-Dawley (SD) rats were selected for this study due to their well-characterized physiology, docile temperament, and widespread use in neurobehavioral and stress-related research, particularly in modeling depressive disorders [23].

In this study, we investigated the potential of AH to modulate stress-induced alterations in glucose regulation, oxidative damage, histopathology of the liver and kidney, and expression of *IL-1β* and *TGF-β1* in vital tissues. We hypothesized that AH supplementation would attenuate hyperglycemia, protect erythrocytes against oxidative hemolysis, ameliorate organ damage, and downregulate inflammatory cytokine expression. Furthermore, we examined whether combining AH with AMT would produce additive or synergistic effects. Our findings aim to expand current knowledge on the therapeutic role of AH as a natural adjunctive intervention for stress-related metabolic and inflammatory dysfunction.

## 2. Methodology

### 2.1. Chemicals

Chemicals were obtained from Sigma-Aldrich Company Ltd., Gillingham, UK, unless otherwise stated.

### 2.2. Honey Collection

AH was collected in Kluang, Johor, Malaysia, in July 2017, corresponding to the dry season when Acacia species are in full bloom and widely maintained by local beekeepers. The honey was freshly obtained from local apiaries within one to three days after harvesting and stored at 4 °C until further use. The honey was produced by the domesticated honeybee species *Apis mellifera*. Our study shows that AH contained *Robinia pseudoacacia* as predominant pollen [14]. The AH sample was not commercially purchased but collected directly from beekeepers to ensure authenticity and freshness.

### 2.3. Housing and Husbandry of Rats

The in vivo study was conducted with slight modifications based on the protocol described by Abidin et al. [24]. All animals used in this study were male Sprague-Dawley (SD) rats bred in-house at the Laboratory Animal Facility and Management (LAFAM), Universiti Teknologi MARA (UiTM). Male rats aged 8 weeks (150–170 g) were used to minimize hormonal variability associated with the estrous cycle in females, which can influence stress responses and behavioral outcomes [25]. The rats were healthy, immunocompetent, and non-genetically modified, with no prior exposure to experimental procedures before the study.

All experimental procedures were approved by the Animal Research and Ethics Committee of Universiti Teknologi MARA (UiTM), Puncak Alam Campus, Faculty of Pharmacy [UiTM CARE:237/2/2018 (6 April 2018)]. Prior to the experiment, the rats underwent a 7-day acclimatization period (3rd-floor LAFAM). Before the experiments, rats were individually caged and fed rat chow and reverse osmosis water (RO) ad libitum while kept under standard laboratory conditions (24 °C ± 1, 45% ± 15 relative humidity and 12 h light/dark cycle). To minimize pain, suffering, and distress, all animals were housed in standard cages with environmental enrichment, including nesting materials and tunnels (tissue roll). Gentle handling techniques were used throughout the study.

### 2.4. Stress-Depression Model

The sample size for the animal study was determined using G*Power software (version 3.1.9.2) based on a one-way ANOVA (fixed effects, omnibus test). The parameters used were an effect size (f) of 0.8329931, significance level (α) of 0.05, statistical power (1–β) of 0.95, and seven experimental groups. Based on these inputs, the total required sample size was calculated to be 42 animals, ensuring an actual power of 0.9767. All animals were retained throughout the experimental procedures and included in the final data analysis.

Forty-two male Sprague-Dawley rats were randomly assigned into seven groups with six rats in each group: (1) normal control (NC), (2) AH (2.25 mg/kg), (3) amitriptyline (AMT, 0.1 mg/kg), (4) stress-induced (S), (5) stress-induced with AH (S + AH), (6) stress-induced with AMT (S + AMT, positive control), and (7) stress-induced with a combination of AH and AMT (S + AH + AMT). All doses were adapted from our preliminary studies by Abidin [24] and Samat [15]. AH and AMT were freshly dissolved in RO ad libitum and administered to the rats.

The experimental timeline was structured to evaluate the glucose level in rats at day 0 and day 30. From day 1 to day 28, rats received daily oral gavage of the respective treatments (AH, AMT, or combination) while being concurrently exposed to CUMS. The CUMS protocol included a randomized schedule of stressors such as overnight illumination, cage tilting, forced swimming, white noise, damp bedding, and restricted movement and rest (Table 1). On day 29, the rats undergone open field test. On day 30, all animals were euthanized via intraperitoneal injection of sodium pentobarbital overdose. Blood samples were collected through cardiac puncture for analysis of blood glucose levels and erythrocyte hemolysis.

### 2.5. Hemolysis Assay

The isolation of erythrocytes for hemolysis assay using hydrogen peroxide (H_2_O_2_) was conducted per Okoko and Ere [27] with slight modification. Briefly, blood was collected into EDTA tubes and centrifuged at 4000 rpm for 10 min at 4 °C. The plasma was discarded, and the erythrocytes were washed three times with phosphate-buffered saline (PBS, 0.2 M, pH 7.4). The resulting packed cells were either stored at −80 °C or immediately used to prepare a 5% erythrocyte suspension (ES) by mixing 50 μL of packed cells with 950 μL of PBS. For the assay, 1 mL of ES was mixed with 1 mL of PBS, with or without 0.5 mL of 10 mM H_2_O_2_, and incubated at 37 °C for 3 h. After incubation, 6 mL of PBS was added to each tube, followed by centrifugation at 2000× *g* for 10 min at 4 °C. The absorbance of the mixture was analyzed at 540 nm using a SmartSpecTM Plus spectrophotometer (Bio-Rad, Hercules, CA, USA).

### 2.6. Histopathology

All animals underwent gross necropsy, followed by histopathological examination of the kidney and liver tissues. Briefly, fixed liver and kidney tissues (10% buffered formalin for 24 h) were embedded in paraffin, sectioned (Leica, Wetzler, Germany), dried, and stained with hematoxylin-eosin (H & E) using an automatic tissue stainer (Leica, Wetzler, Germany). After overnight incubation, the stained slides were mounted using DPX (Sigma-Aldrich, St. Louis, MO, USA) and viewed under a light microscope (Leica, Wetzler, Germany) using randomization method. Changes observed in liver and kidney tissue sections were graded using a semiquantitative lesion scoring system. Scores were assigned as follows: 0 for no observable changes, 1 for mild changes, 2 for moderate changes, and 3 for severe histopathological alterations. For each group, the mean lesion score was calculated from six animals, with one representative tissue section analysed per organ per animal. All assessments were performed in a blinded manner by an experienced histopathologist to ensure consistency and minimize bias.

### 2.7. Quantitative Real-Time PCR

Total RNA was extracted from approximately 30 mg of tissue samples (ileum, caecum and hypothalamus) using the SV Total RNA Isolation System (Promega, Madison, WI, USA, Cat # Z3105). RNA purity and quantity were measured by NanoDrop Lite Spectrophotometer (Thermo Scientific, Waltham, MA, USA) (A260/280, Eppendorf; 1.8–2.2). cDNA (2 µL) was synthesized with a GoScriptTM Reverse Transcription Mix, Oligo (dT) (Promega, USA, Cat # A2791). Quantitative real-time PCR was performed using a GoTaq^®^ qPCR Master Mix (Promega, USA, Cat # A6001). RT-qPCR assays were run on a Rotor Gen Q 5 plex HRM platform (Qiagen, Hilden, Germany) in 36-tube over 40 cycles of 95 °C for 15 s and 60 °C for 1 min by holding temperature of 95 °C for 2 min before cycle starts. Rotor gene Q series software v 2.3.5 was used to acquire the threshold cycle (Ct) values and normalized to those of *GAPDH*. Fold differences were determined using the 2^−ΔΔCT^. The primer sequences utilized in this study are presented in Table 2.

### 2.8. Statistical Analysis

Data were presented as mean ± standard error of the mean (SEM) from at least three independent experiments. Data normality was assessed using the Shapiro–Wilk test. When assumptions of normality were violated, appropriate non-parametric tests were applied. However, as all data were normally distributed, parametric test were used throughout the study. Blood glucose levels were analyzed using both paired-*t*-test and one-way analysis of variance (ANOVA), while lesion scores were analyzed using two-way ANOVA. Data for hemolysis and gene expression were analyzed using one-way ANOVA with Bonferroni post hoc test. Differences between group means were considered statistically significant at *p* < 0.05. All statistical analyses were performed using GraphPad Prism version 8.0 (GraphPad Software, San Diego, CA, USA).

## 3. Results and Discussion

Stress-related depression is a multifactorial disorder involving neuroendocrine and metabolic dysregulation. The chronic unpredictable mild stress (CUMS) model is widely used to mimic human depression. The successful establishment of the CUMS model is typically validated through behavioral and biochemical markers that reflect depressive-like states. In our preliminary study [28,29], two core indicators were employed: the sucrose preference test (SPT) and serum cortisol levels. The SPT is a widely recognized behavioral assay for assessing anhedonia, a key symptom of depression, where reduced sucrose consumption indicates diminished reward sensitivity [30,31]. Concurrently, elevated cortisol levels serve as a biochemical marker of HPA axis hyperactivity, which is commonly observed in stress-induced depression [32]. These indicators are consistent with established protocols for validating CUMS models, which often include behavioral tests such as the open field test, alongside biochemical assays.

**Figure 2:**
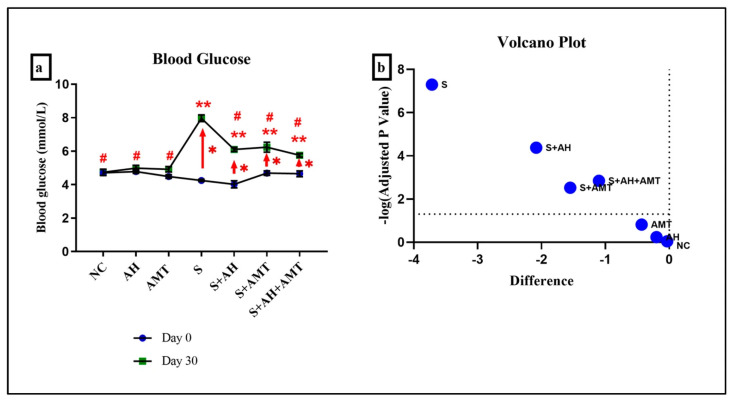
(**a**) Blood glucose level in rats were measured on day 0 and day 30 in all groups. Comparison was performed using Paired-*t*-test. (**b**) The results were visualized in a volcano plot. The higher plot values observed in the S group compared to other groups indicate the most significant changes occured in the S group. * indicates statistical significance (*p* < 0.05) comparison between day 0 and day 30 within the same group (red arrow, Paired-*t*-test). ** indicates statistical significance (*p* < 0.05) comparison with NC group on Day 30 (one-way ANOVA), # indicates statistical significance (*p* < 0.05) compared with S group comparison using one-way ANOVA, n = 6 independent observations.

CUMS activates the HPA axis, leading to elevated blood glucose levels due to impaired glucose homeostasis and stress-induced hyperglycemia [33]. Figure 2 illustrates the blood glucose levels in rats on day 0 and day 30 across all experimental groups. Since AH contains natural sugars that could potentially influence blood glucose levels, this parameter was monitored to evaluate its metabolic impact. The data were collected to determine whether AH supplementation, particularly under stress conditions, contributes to hyperglycemia or exhibits a regulatory effect on glucose homeostasis.

Before the experiment (day 0), no significant differences in blood glucose were observed among the group (*p* > 0.05), indicating comparable baseline metabolic states. Interestingly, on day 30, rats supplemented with AH alone (AH group) showed no significant change in blood levels (*p* = 0.58), suggesting that AH does not induce hyperglycemia and may be metabolically safe. This finding is consistent with previous research showing that honey, particularly Acacia honey, has a low glycemic index and may improve glycemic control due to its high fructose content and antioxidant properties [34,35].

In contrast, the stress-induced group (S) demonstrated a significant elevation in blood glucose at the end of experiment (7.97 ± 0.20 mmol/L) compared with day 0 (4.25 mmol/L ± 0.07, *p* = 0.00) (Figure 2, red arrow). This confirms that CUMS model disrupts glucose homeostasis, leading to a hyperglycaemic state. The rise in blood glucose is consistent with the well-documented role of stress in activating the HPA axis, resulting in increased glucocorticoid secretion, enhanced hepatic gluconeogenesis, and reduced insulin sensitivity [1]. These metabolic alterations explain the significant difference between pre- and post-supplementation values observed in the stress group.

Interestingly, on day 30, rats supplemented with Malaysian AH (S + AH) showed a reduction in blood glucose levels to 6.10 mmol/L ± 0.12, while AMT treatment (S + AMT) produced a comparable reduction to 6.23 mmol/L ± 0.30, *p* = 0.00. Both interventions significantly mitigated the hyperglycaemic response associated with stress exposure. Importantly, the combined supplementation of AH and AMT (S + AH + AMT) resulted in the greatest reduction (5.75 mmol/L ± 0.11, *p* = 0.00). The values approaching closer to those of non-stressed controls. This suggests a potential synergistic effect between natural supplementation and pharmacological treatment in improving metabolic outcomes under stress conditions.

The glucose-lowering effect of AH may be attributed to its high content of phenolic compounds and flavonoids, which are known to enhance antioxidant defenses, protect pancreatic β-cell integrity, and improve peripheral insulin sensitivity [9,11,14]. Previous in vivo studies have shown that honey supplementation attenuates oxidative stress and hyperglycaemia in diabetic models, supporting its potential therapeutic role beyond basic nutrition [9,11]. In addition, a clinical trial in humans demonstrated that honey consumption stabilized fasting blood sugar and enhanced antioxidant status, further reinforcing its beneficial metabolic properties [10].

On the other hand, AMT, a tricyclic antidepressant, exerts its effect mainly through modulation of serotonergic and noradrenergic neurotransmission. Its impact on glucose regulation is likely indirect, mediated by a reduction in the neuroendocrine burden of chronic stress rather than direct action on glucose metabolism [8]. However, when combined with AH, the overall improvement in blood glucose suggests complementary pathways. Honey may reduce oxidative stress and inflammation [36,37], while AMT alleviates stress-related neurochemical changes via TrkA signaling pathways [38] and enhancing glycaemic control [38,39].

Previous studies have demonstrated a link between blood glucose dysregulation and hemolysis [40]. Stress-induced hyperglycemia elevates circulating glucose, facilitating erythrocyte glycation and oxidative stress. These processes compromise membrane integrity, reduce cellular deformability, and promote eryptosis, ultimately leading to hemolysis under stress conditions [40].

**Figure 3:**
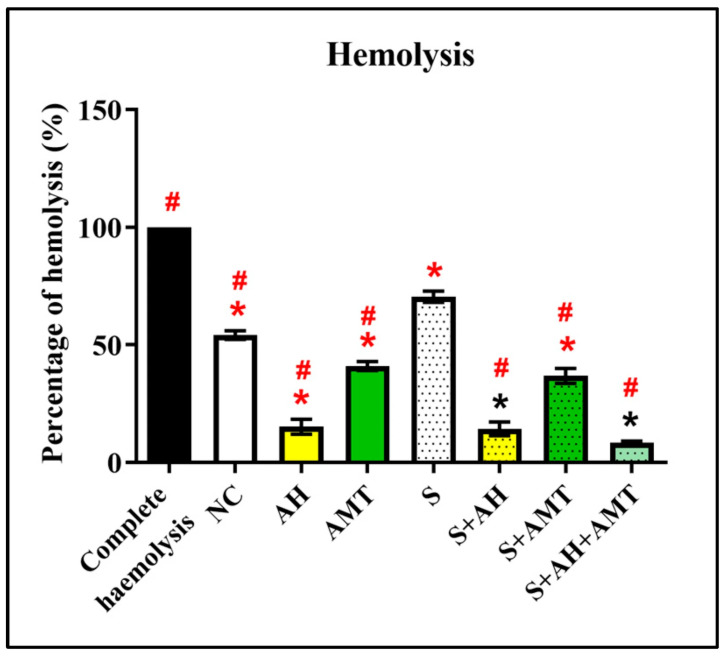
Percentage of blood hemolysis induced by H_2_O_2_ on day 30. Acacia honey protects the blood against H_2_O_2_-induced lysis, indicated by a low percentage of hemolysis in the S + AH group compared to the S group. * indicates statistical significance (*p* < 0.05) compared with the NC group. # indicates statistical significance (*p* < 0.05) compared with S group comparison using one-way ANOVA, with Bonferroni post hoc test, n = 6 independent observations.

An in vitro blood hemolysis assay was conducted at the end of the in vivo study to evaluate the protective effect of AH against oxidative stress-induced hemolysis (Figure 3). All treatment groups [NC (81.00% ± 4.64), AH (15.15% ± 3.21), AMT (40.95% ± 1.91), S (70.48% ± 2.41), S + AH (14.30% ± 2.96), S + AMT (36.80% ± 3.18), and S + AH + AMT (8.45% ± 0.62)] showed a significant reduction in hemolysis compared to the complete hemolysis control group (100% ± 0.00, *p* = 0.00). Particularly, the S + AH, S + AMT, and S + AH + AMT groups exhibited significantly lower hemolysis compared to the S group (*p* = 0.00), indicating that AH, alone or in combination with AMT, effectively mitigates H_2_O_2_-induced erythrocyte damage under stress conditions. This is due to high phenolic and flavonoid content in AH, as demonstrated in our previous study [14].

The current study was consistent with the previous finding, in which healthy rats that supplemented with AH (AH group) were found to exhibit low blood hemolysis [41]. As the pattern was similar in the rats-induced stress and supplemented with AH (S + AH group), these further confirm the protective effect of AH was by inhibiting blood hemolysis-induced with H_2_O_2_. The protective effect of AH was due to the binding of the flavonoids to the blood cell membrane, preventing H_2_O_2_ from attacking the erythrocyte membrane [41]. Particularly, AH was demonstrated to have a high scavenging capacity [14]. A clinical trial involving participants supplemented with honey for two weeks reported increased levels of antioxidant biomarkers, including blood vitamin C, beta-carotene, and glutathione reductase, as well as improvements in serum iron, hematological indices, and trace elements [10,36].

Hemolysis during stress releases free hemoglobin and heme, overwhelming scavenger systems and promoting systemic oxidative stress [42]. Excess iron from hemolysis catalyzes reactive oxygen species formation, aggravating hepatocellular injury [43]. Histopathological findings, including vacuolar degeneration and inflammatory infiltration, reflect this oxidative burden and link erythrocyte destruction to liver pathology under stress conditions [44].

**Figure 4:**
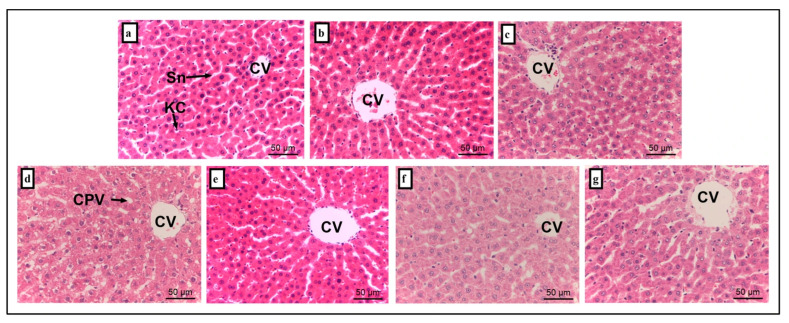
Histological sections of liver tissue. Representative liver sections from each experimental group (n = 6): (**a**) normal control, (**b**) Acacia honey, (**c**) amitriptyline, (**d**) stress-induced, (**e**) stress treated with Acacia honey, (**f**) stress treated with amitriptyline, and (**g**) stress treated with a combination of Acacia honey and amitriptyline. CV, central vein; Sn, sinusoidal; KC, Kupffer cell; CPV, cytoplasmic vacuolation. Scale bar: 50 µm.

**Figure 5:**
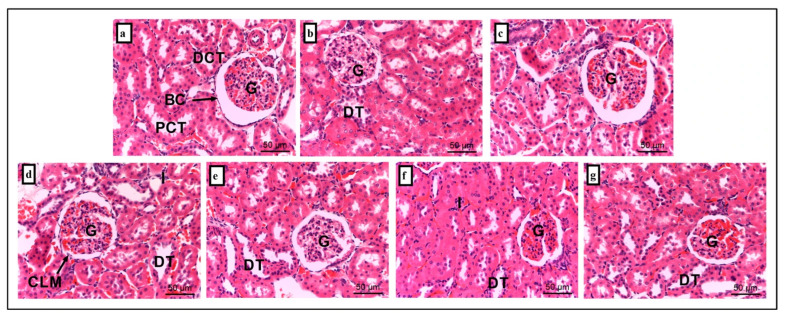
Histological section of kidney tissue. It was consists of groups (**a**) NC, (**b**) acacia honey, (**c**) amitriptyline, (**d**) stress-induced, (**e**) stress treated with Acacia honey, (**f**) stress treated with amitriptyline, and (**g**) stress treated with a combination of Acacia honey and amitriptyline. G, glomerulus; BC, bowman’s capsule; PCT, proximal convoluted tubules; DCT, distal convoluted tubules; CLM, cell lining missing; DT, dilated intertubular capillaries; I, inflammation. Scale bar: 50 µm. n = 6 independent observations.

Histopathological changes in the liver and kidney tissues are summarized in Figure 4 and Figure 5, with corresponding lesion scores presented in Table 3. Liver sections from the NC (Figure 4a) and AH group (Figure 4b) exhibited normal histoarchitecture, including intact central vein (CV), sinusoidal (Sn), and Kupffer cell (KC) (lession score, 0.00 ± 0.00). In contrast, liver tissues from the AMT (Figure 4c) and S group (Figure 4d) showed cytoplasmic vacuolation with the lesion score of 1.20 ± 0.37 and 1.40 ± 0.25 when compared to the NC group (*p* = 0.00), respectively. Notably, the S + AH (Figure 4e; 0.40 ± 0.25) and S + AH + AMT (Figure 4g; 0.00 ± 0.00) groups demonstrated no significant histological alterations compared to the NC group (*p* = 0.99). A significant reduction in cytoplasmic vacuolation was observed in both S + AH and S + AH + AMT groups relative to the S group, indicating the hepatoprotective effect of AH, particularly when combined with AMT.

Histological liver examination of stress rats reveals prominent cytoplasmic vacuolation. A previous study has reported that an increase in serum AST [45], ALT [45] and GGT levels reflects the loss of structural integrity of the liver [46], which corroborated the current findings. AST is released into the bloodstream due to hepatocellular degeneration and necrotic changes leading to elevated serum AST levels [46]. Although mild cytoplasmic vacuolation was observed in the liver tissues of stress-induced rats, no signs of hepatocellular degeneration or necrosis were detected. Cytoplasmic vacuolization in mammalian cells can be transient or irreversible. The transient vacuolization can only be observed during exposure to stressors and may be reversibly affects the cell cycle and migration. Meanwhile, cytoplasmic vacuolation was not observed in the stress rats supplemented with AH. These findings suggest AH may have protective effects towards stress-induced hepatocellular damage, likely through its antioxidant and anti-inflammatory properties.

Similarly, free hemoglobin and iron deposition in renal tissue induce oxidative stress and tubular damage, contributing to acute kidney injury [47]. Persistent hemolysis can further promote renal fibrosis and chronic kidney disease through iron-mediated oxidative pathways [48]. Subsequently, kidney sections were examined to assess the structural effects of stress on renal tissue. Histology of kidney tissue for the NC group (Figure 5a) reveals intact renal architecture, including well-preserved glomerulus (G), Bowman’s capsule (BC), proximal convoluted tubules (PCT), and distal convoluted tubules (DCT). However, the S group (Figure 5d) exhibited significant pathological changes, including decreased of tubular cell lining (1.200 ± 0.374), dilated intertubular capillaries (1.400 ± 0.510), and inflammation (2.200 ± 0.200) when compared to the NC group (0.000 ± 0.000, *p* = 0.00). Supplementation with AH (S + AH group; Figure 5e), AMT (S + AMT group; Figure 5f), and their combination (S + AH + AMT group; Figure 5g) significantly decreased tubular cell lining and inflammation (Table 3) when compared to S group only (*p* = 0.00).

In particular, the histology of kidney tissue of the stress rats demonstrated dilated intertubular capillaries, inflammation and absence of cell lining. Supplementation with AH in stress rats significantly reduced abnormal morphology characteristics. The presence of abnormal structure in stress rats was associated with the overproduction of reactive oxygen species [49], leading to activation of IL-1 [2], IL-6 [2], COX-2 [50] and TNF-α [50]. The protective effect of honey on renal tubular has been shown previously against cadmium through honey’s ability to reduce lipid peroxidation and increase tissue levels of glutathione and glutathione peroxidase activity [49]. These findings suggest that AH may have protective effects against stress-induced hepatocellular and kidney damage. The protective effect was observed to be further enhanced with the co-supplementation with both AH and AMT.

Interleukin-1β (IL-1β) plays a critical role in mediating immunological, behavioral, and neuroendocrine responses to infection and inflammation. It is also implicated in the activation of the HPA axis during stress. Therefore, this study investigated *IL-1β* gene expression on three HPA axis-related organs: the ileum, caecum and hypothalamus. Real-time PCR was employed to assess the transcriptional regulation of pro-inflammatory cytokines, *IL-1β* in the ileum, caecum, and hypothalamus.

**Figure 6:**
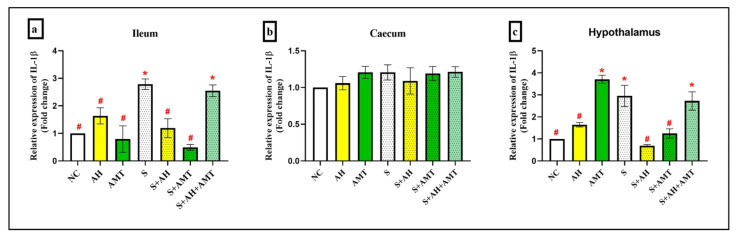
*IL-1β* gene expressions in (**a**) ileum, (**b**) caecum, and (**c**) hypothalamus. *, shows the comparison between NC group and other groups indicates statistical significance (*p* < 0.05); #, the comparison between stress rats (S) with other groups indicates statistical significance (*p* < 0.05), comparison using one-way ANOVA, Bonferroni post hoc test, n = 3 independent observations.

In the ileum (Figure 6a), *IL-1β* expression is significantly upregulated in the S (2.79-fold ± 0.19, *p* = 0.01) and S + AH + AMT groups (2.55-fold ± 0.21, *p* = 0.03) compared to the NC group (1.00-fold ± 0.00). No significant changes were observed in the AH (1.64-fold ± 0.29, *p* = 0.99), AMT (0.79-fold ± 0.48, *p* = 0.99), S + AH (1.19-fold ± 0.33, *p* = 0.99) and S + AMT groups (0.49-fold ± 0.10, *p* = 0.99). In contrast, treating stress rats with AH (S + AH, *p* = 0.02) and AMT (S + AMT, *p* = 0.00) downregulated the *IL-1β* gene expression.

In contrast, no significant changes were observed in the caecum sample (Figure 6b) for group of AH (1.06-fold ± 0.09, *p* = 1.00), AMT (1.21-fold ± 0.08, *p* = 0.54), S (1.21-fold ± 0.10, *p* = 0.54), S + AH (1.09-fold ± 0.18, *p* = 0.97) S + AMT (1.19-fold ± 0.09, *p* = 0.60) and S + AH + AMT (1.21-fold ± 0.07, *p* = 0.51) compared to NC group (1.00-fold ± 0.00). *IL-1β* was not modulated in the caecum during stress-induced depression in rats, possibly due to regional immune tolerance and lower microbial stimulation compared to the ileum. The ileum includes higher microbial density and immune cell activity, as previously reported in TH17 cell expansion that enhance IL-1β signalling via microbiota-derived cues and NF-κB activation [51].

In this study, *IL-1β* was shown to be upregulated in the stress rats’ ileum, which is in line with other studies [52,53]. Intestine tissues from mice with colitis were reported to have higher amounts of *IL-1β* mRNA, which inhibits the development of intestinal inflammation by reducing occludin expression by enterocytes and therefore increasing tight junction permeability [53]. A similar report was demonstrated by Uchakin et al. [52] during the academic examination that increases IFN-γ, IL-2, IL-1α and IL-1β in the serum of stress medical student and the expression reduced after 2 days post examination.

In the hypothalamus, *IL-1β* gene expression was significantly upregulated in the AMT (3.71-fold ± 0.18, *p* = 0.00), S (2.95-fold ± 0.48, *p* = 0.00) and S + AH + AMT (2.73-fold ± 0.41, *p* = 0.01) groups compared to the NC group (1.00-fold ± 0.00). Contrarily, treatment with AH (S + AH; 0.70-fold ± 0.04, *p* = 0.00) and AMT (S + AMT; 1.25-fold ± 0.21, *p* = 0.01) significantly downregulated *IL-1β* expression compared to the S group (Figure 6c).

In this study, *IL-1β* was shown to be upregulated in the stress rats’ hypothalamus, which is in line with other studies [52,53]. In addition, *IL-1β* mRNA levels in the hypothalamus in intact rats were increased by LPS injection [54]. c-FOS is an immediate early gene commonly used as a marker of neuronal activation in response to various stimuli. During stress, the increment of *IL-1β* during stress might increase action potential firing in vagal afferents, induced C-FOS expression in the nucleus tractus solitarii, the central projection area of vagal afferent nerve fibres the medullary brainstem, and sensitized vagal afferent pathways to gastric acid [55].

The increase in *IL-1β* expression observed during stress was significantly downregulated following supplementation with AH, AMT or their combination. This suggests that both AH and AMT exert anti-inflammatory effects, potentially through modulation of the gut–brain axis. Honey has been shown to influence brain function via the gut–brain axis by promoting the growth of beneficial gut bacteria, such as Bifidobacteria, which in turn reduce oxidative stress and inflammation in the gut. These bacteria produce short-chain fatty acids [56,57], such as butyrate and propionate, which have been reported to enhance intestinal barrier integrity, modulate immune responses, and influence neuroinflammation through signaling pathways that affect the central nervous system [56]. In particular, these events result in appropriate myelination of neurons, blood–brain barrier repair, decreased production of inflammatory cytokines (IL-1β, TNF-α, and BDNF), and mediated restoration of synaptic plasticity in the brain increase in memory [58].

IL-1β and TGF-β1 exhibit a synergistic role in stress-induced pathology [59]. IL-1β amplifies inflammation and oxidative stress, while TGF-β1 drives fibrotic remodeling [60]. Their combined activation promotes epithelial–mesenchymal transition and extracellular matrix deposition, accelerating organ dysfunction under chronic stress conditions [59,60].

**Figure 7:**
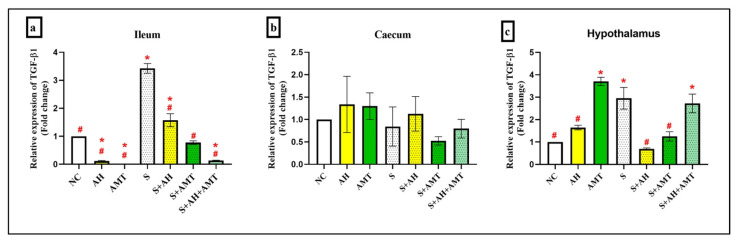
*TGF-β1* gene expressions in ileum (**a**), caecum (**b**), and hypothalamus (**c**). *, shows the comparison between NC group and other groups indicates statistical significance (*p* < 0.05); #, the comparison between stress rats (S) group with other groups indicates statistical significance (*p* < 0.05), comparison using one-way ANOVA, Bonferroni post hoc test, n = 3 independent observations.

Based on Figure 7, the expression of *TGF-β1* gene shows distinct patterns across the ileum, caecum, and hypothalamus. *TGF-β1* expression in the ileum (Figure 7a) was significantly downregulated in AH group (0.11-fold ± 0.02), followed by S + AH + AMT (0.14-fold ± 0.01) and AMT group (0.00-fold ± 0.00) compared to the NC group (1.00-fold ± 0.00, *p* = 0.00). Stress alone (S group; 3.42-fold ± 0.17) significantly upregulated the *TGF-β1* gene expression (3.43-fold ± 0.17), while S + AH (1.57-fold ± 0.23) and S + AMT (0.77-fold ± 0.06) showed reduction in *TGF-β1* expression (*p* = 0.00).

The significant downregulation of *TGF-β1* in the ileum following AH supplementation suggests honey anti-inflammatory potential in stress-induced depression. *TGF-β1* is known to mediate mucosal immunity and fibrosis in the gut, and its overexpression under stress may exacerbate intestinal inflammation. AH appears to restore immune homeostasis, possibly via modulation of Smad-dependent pathways [61,62]. The combination of AH and AMT also reduced *TGF-β1*, indicating synergistic effects. These findings align with studies showing that natural antioxidants can suppress *TGF-β1* signaling and improve gut barrier integrity [63].

In the caecum (Figure 7b), no significant changes were observed in TGF-β1 expression in group of AH (1.34-fold ± 0.63, *p* = 0.96), AMT (1.30-fold ± 0.30, *p* = 0.98), S (0.84-fold ± 0.44, *p* = 1.00), S + AH (1.13-fold ± 0.39, *p* = 1.00), S + AMT (0.52-fold ± 0.09, *p* = 0.84), and S + AH + AMT (0.80-fold ± 0.21, *p* = 1.00) compared to NC group (1.00-fold ± 0.00). In contrast to ileum, *TGF-β1* expression in the caecum remained relatively unchanged across all groups, suggesting regional specificity in cytokine regulation. The caecum’s microbial richness may buffer against stress-induced cytokine fluctuations, maintaining immune equilibrium [64]. Previous studies have shown that *TGF-β1* plays a role in maintaining mucosal tolerance and preventing excessive immune activation [65]. The lack of significant modulation by AH or AMT implies that therapeutic effects may be more pronounced in regions with active inflammation or epithelial turnover.

Under stress, elevated TGF-β1 contributes to brain inflammation, fibrosis, and immune dysregulation. In the hypothalamus (Figure 7c), *TGF-β1* expression was significantly increased in AMT (3.71-fold ± 0.19), S (2.95-fold ± 0.48), and S + AH + AMT group (2.73-fold ± 0.41) compared to NC group (1.00-fold ± 0.00, *p* = 0.00). This effect was consistent with its role in neuroinflammation and HPA axis dysregulation [66]. High level of *TGF-β1* has been reported for neuroprotective and anti-inflammatory effects [67] as well as involve in microglial activation and depressive-like behaviors, and its modulation may influence neuroimmune signaling [67].

During stress, AH (S + AH; 0.70-fold ± 0.04, *p* = 0.00) and AMT (S + AMT; 1.25-fold ± 0.21, *p* = 0.00) had showed reduction in *TGF-β1* levels. Malaysian AH contains phenolic compounds and flavonoids that exert antioxidant and anti-inflammatory effects, modulating cytokine expression. Honey downregulates *TGF-β1* by modulating NF-κB and MAPK pathways [68], which are upstream regulators of *TGF-β1*. The reduction in *TGF-β1* expression by AMT was due to the inhibiting of microglial activation in the brain, which is a major source of TGF-β1 during neuroinflammation [67], modulating calcium signaling and sigma-1 receptor activity, which affects cytokine release in immune cells [39] and reducing oxidative stress and neuroinflammation, thereby indirectly suppressing *TGF-β1* transcription [69].

In addition, supplementation of AMT alone increased the *IL-1β* (Figure 6c) and *TGF-β1* expression (Figure 7c). Although AMT is known for its anti-inflammatory effects, its impact on central cytokine expression can be context dependent. In non-stressed rats, AMT may modulate monoaminergic neurotransmission, leading to microglial activation and increased IL-1βa and *TGF-β1* expression in the hypothalamus.

## 4. Conclusions

**Figure 8:**
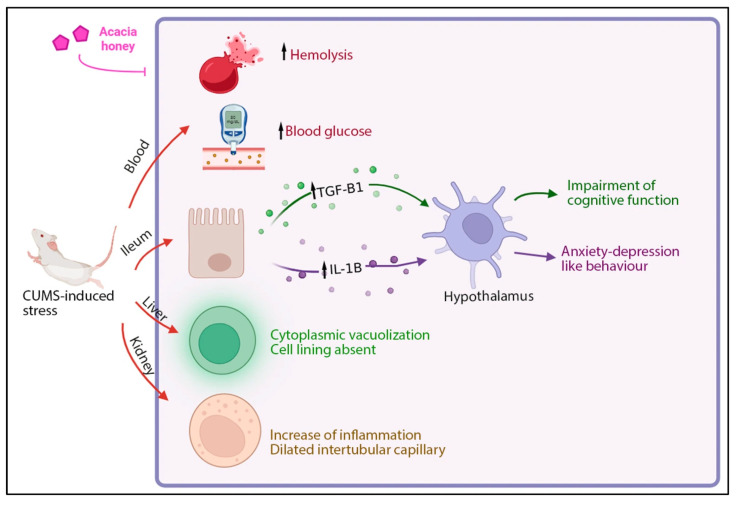
Summary of AH’s effect on hemolysis, blood glucose, *IL-1β* and *TGF-β1* gene expression in the ileum and hypothalamus, as well as structural alteration in kidney and liver under stress conditions. The figure illustrates that CUMS-induced stress increases hemolysis, blood glucose levels, inflammation (black arrow), and anxiety- and depression-like behaviors, while impairing cognitive function and damaging multiple organs including the liver and kidney. Supplementation with AH effectively blocks these effects, reducing inflammation and behavioral symptoms, and restoring physiological and cellular integrity. Image created using Biorender.com.

The current study demonstrates that supplementation with AH significantly modulates stress-induced metabolic, oxidative, and inflammatory disturbances (Figure 8). Chronic unpredictable mild stress induced hyperglycemia, increased erythrocyte hemolysis, hepatic vacuolation, renal tubular injury, and upregulation of pro-inflammatory cytokines (*IL-1β* and *TGF-β1*) in the ileum, caecum, and hypothalamus. Supplementation with AH markedly reduced blood glucose, protected erythrocytes against H_2_O_2_-induced hemolysis, and ameliorated histopathological changes in both liver and kidney. In parallel, AH downregulated *IL-1β* and *TGF-β1* expression in stress-related tissues, reflecting anti-inflammatory modulation.

AMT, used as the pharmacological control, improved several parameters, but the combined treatment with AH and AMT produced the most profound effects, suggesting a synergistic interaction. The mechanisms underlying these protective outcomes may involve the high phenolic and flavonoid content of AH, which can enhance antioxidant defenses, reduce oxidative stress, and improve insulin sensitivity. In addition, the anti-inflammatory and membrane-stabilizing properties of honey further contribute to its protective role.

Findings from this study using male Sprague-Dawley rats in the CUMS model are relevant to human depression, as the model replicates key behavioral and neuroinflammatory features of the disorder. Despite species differences, the results offer translational insights into stress-related pathophysiology and may inform therapeutic strategies for human depressive conditions.

A key limitation of this study is the complexity of stress tolerance mechanisms in mammals which may involve adaptive responses at multiple biological levels. Beyond metabolomic shifts, stress-induced tolerance is mediated by receptor activation, protein signaling cascades, and serum biomolecule modulation. These processes orchestrate transcriptomic and proteomic reprogramming, influencing metabolic pathways such as glucose and amino acid metabolism. A systemic biology approach is essential to elucidate the interplay between stress-related transcription factors, receptor-mediated signaling, and serum biomarkers. Future studies should expand to clinical trials to confirm translational relevance, establish dose–response relationships, and explore the molecular mechanisms linking honey’s antioxidant compounds with neuroendocrine regulation.

## Figures and Tables

**Table 1:** Chronic unpredictable mild stress procedure.

No	Stressor *	Procedure
1	Overnight illumination	Illumination of cages containing rats for 24 h
2	Cage tilting	The cage is tilted for 24 h
3	Forced swimming	The rats were forced to swim for 5 min
4	White noise	The rats were introduced to intermittent 60 dB white noise stress for 2 h
5	Damp bedding	The bedding was damped for 24 h
6	Restricted movement	The rats will live in a closed compartment system for 2 h
7	Rest	No stressor was given to the rats

* All stressors were conducted according to Abidin et al. (2017) [24] and Zhang et al. (2017) [26]. Stressors were randomised using Research Randomiser software version 4.0. One random stressor out of six selected stressors will be introduced daily to induce stress in rats.

**Table 2:** Primer Sequences.

Primer Name	Primer Sequence (5′ to 3′)
*F_GAPDH*	AGTGCCAGCCTCGTCTCATA
*R_GAPDH*	GATGGTGATGGGTTTCCCGT
*F_Interleukin 1 beta*	GACTTCACCATGGAACCCGT
*R_Interleukin 1 beta*	GGAGACTGCCCATTCTCGAC
*F_TGFβ1*	CGTCAGACATTCGGGAAGCA
*R_TGFβ1*	TGCCGTACAACTCCAGTGAC

**Table 3:** Lesion scores of kidneys and liver tissues.

Group	Liver		Kidney	
	Cytoplasmic Vacuolation	Cell Lining Absence	Dilated Intertubular Capillaries	Inflammation
NC	0.00 ± 0.00	0.00 ± 0.00	0.00 ± 0.00	0.00 ± 0.00
AH	0.00 ± 0.00	0.00 ± 0.00	0.20 ± 0.20	0.20 ± 0.20
AMT	1.20 ± 0.37 *	0.20 ± 0.20	0.60 ± 0.25	0.60 ± 0.40
S	1.40 ± 0.25 *	1.20 ± 0.37 *	1.40 ± 0.51 *	2.20 ± 0.20 *
S + AH	0.40± 0.25 #	0.20 ± 0.20 #	0.60 ± 0.25	0.60 ± 0.25 #
S + AMT	1.20 ± 0.20 *	0.20 ± 0.20 #	0.60 ± 0.40	0.60 ± 0.25 #
S + AH + AMT	0.00 ± 0.00 #	0.00 ± 0.00 #	0.80 ± 0.37	0.40 ± 0.25 #

* Indicates statistical significance (*p* < 0.05) compared with NC group. # Indicates statistical significance (*p* < 0.05) compared with the S group, comparison using two-way ANOVA, Bonferroni post hoc test, n = 6 independent observations.

## Data Availability

The original contributions presented in the study are included in the article, further inquiries can be directed to the corresponding author.

## Abbreviations

The following abbreviations are used in this manuscript:

AH	Malaysian Acacia honey
ALT	Alanine aminotransferase
AMT	Amitriptyline
ANOVA	Analysis of variance
AST	Aspartate transaminase
BDNF	Brain-derived neurotrophic factor
BC	Bowman’s capsule
c-FOS	Celular-FOS
CLM	Cell lining missing
COX-2	Cyclooxygenase-2
CPV	Cytoplasmic vacuolation
Ct	Threshold cycle
CUMS	Chronic unpredictable mild stress
CV	Central vein
DCT	Distal convoluted tubules
DNA	Deoxyribonucleic acid
DT	Dilated intertubular capillaries
ES	Erythrocyte suspension
G	Glomerulus
*GAPDH*	Glyceraldehyde 3-phosphate dehydrogenase
GGT	Gamma-glutamyltransferase
GLUT2 receptor	Glucose Transporter Type 2 receptor
GLUT5 receptor	Glucose Transporter Type 5 receptor
h	Hour
H_2_O_2_	Hydrogen peroxide
HPA	Hypothalamic–pituitary–adrenal
H&E	Hematoxylin-eosin
iNOS	Inducible Nitric Oxide Synthase
I	Inflammation
IL-1	Interleukin-1
IL-1α	Interleukin-1 alpha
IL-1β	Interleukin-1 beta
IL-2	Interleukin-2
IL-6	Interleukin-6
IL-10	Interleukin-10
IFN-γ	Interferon gamma
KC	Kupffer cell
LPS	Lipopolysaccharide
LOXs	Lipoxygenases
min	minute
mRNA	Messenger ribonucleic acid
MDD	major depressive disorder
NC	Normal control
NF-κB	Nuclear Factor kappa-light-chain-enhancer of activated B cells
NO	Nitric Oxide
IκBα	Inhibitor of kappa B alpha
PBS	Phosphate-buffered saline
PCT	Proximal convoluted tubules
PDGF	Platelet-Derived Growth Factor
RNA	Ribonucleic acid
RO	Reverse osmosis water
ROS	Reactive oxygen species
S	Stress-induced group
SEM	Standard error of mean
Sn	Sinusoidal
SD	Sprague-Dawley
SPT	Sucrose preference test
TGF-β	Transforming growth factor beta
*TGF-β1*	Transforming growth factor beta 1 gene
TNF-α	Tumor necrosis factor-alpha
